# Supplementary material for: Dissecting the cell cycle regulation, DNA damage sensitivity and lifespan effects of caffeine in fission yeast
Source: Microb Cell. 2025 Jun 24;12:141–56. doi: 10.15698/mic2025.06.852 (PMC12203419; doi:10.15698/mic2025.06.852)
Supplement: Supplementary file 1 [file mic-12-141-s01.pdf]

Dissecting the cell cycle regulation, DNA damage sensitivity and lifespan effects of caffeine in fission yeast

John- Patrick Alao[1], Juhi Kumar[1], Despina Stamataki[2] and Charalampos Rallis[1,*]

[1] Research Centre of Molecular Cell Biology, School of Biological and Behavioural Sciences, Queen Mary University of London, Mile End Road, London, E1 4NS, United Kingdom. [2] The Francis Crick Institute, London, United Kingdom.

⁎ Corresponding Author:
Charalampos Rallis, E-mail: c.rallis@qmul.ac.uk

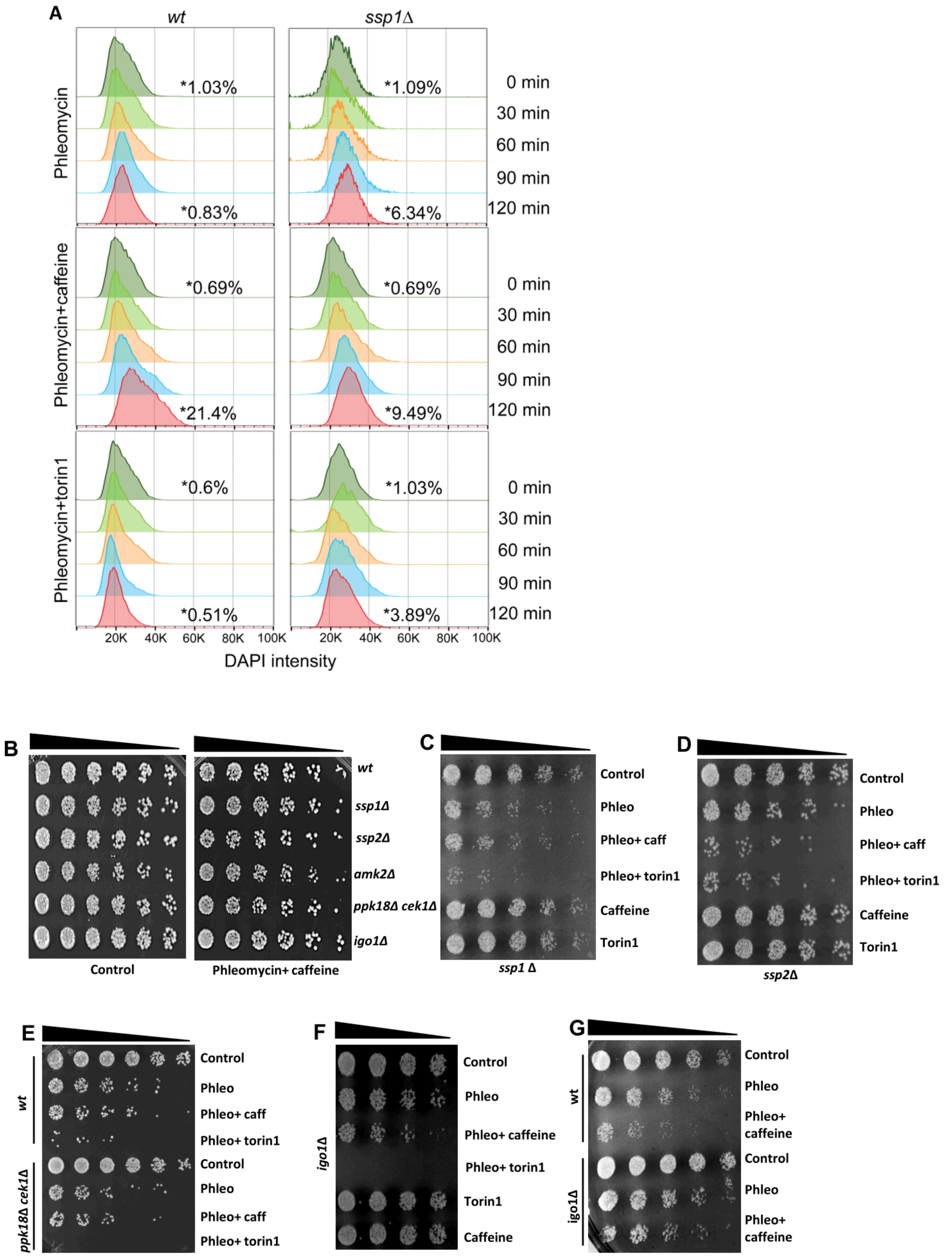

Supplementary Figure S1. (A) FACS analysis of wt and *ssp1* mutant cells following pharmacological treatments in a time course fashion as indicated. *Wt* and *ssp1Δ* strains were exposed to 5 μg/mL phleomycin for 2 h. Samples were then left untreated or co-exposed to 10 mM caffeine or 5 μM Torin1 and harvested every 30 minutes for 2 h. Numbers within the graphs indicate the percentage of cells that pass the 40K DAPI intensity arbitrary cutoff in each case. (B-G) The indicated *wt* and mutant strains were incubated with 5 μg/mL phleomycin alone for 2 h and then for a further 2 h with 10 mM or 5 μM Torin1. They were then serially diluted 3-fold, plated on YES agar and incubated for 3-5 days at 32°C.

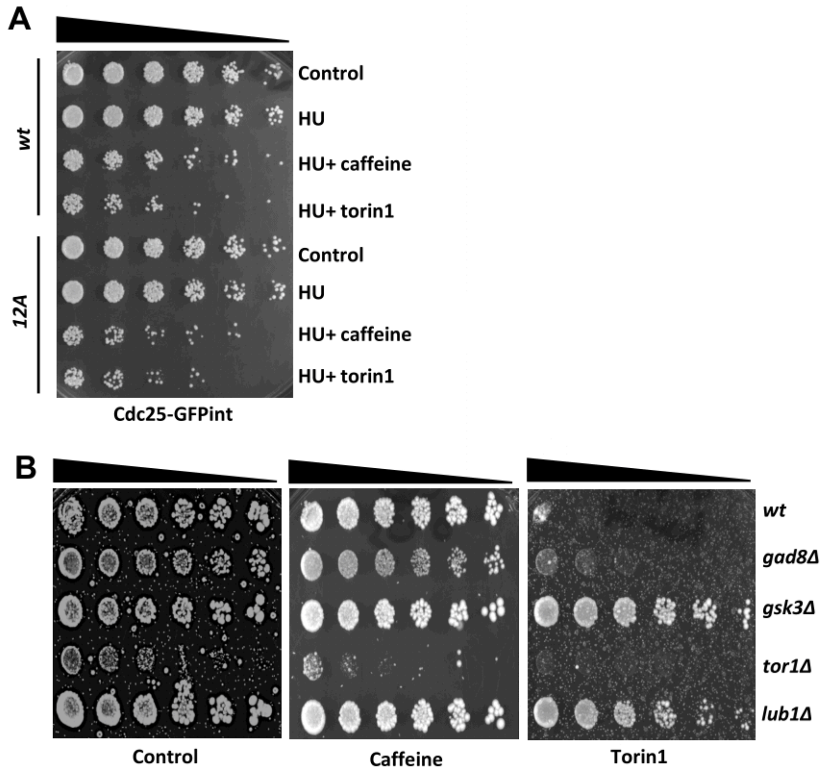

Supplementary Figure S2. Caffeine enhances DNA damage sensitivity independently of its cell cycle effects. (A- E) The indicated wild type and mutant strains were grown to log phase and left untreated or incubated with 5 µg/ mL phleomycin for 2 h. Cultures were then incubated for a further 2 h with or without 10 mM caffeine or 5 µM Torin1 in 10 mL medium for a further 2 h as indicated. Cultures were adjusted for cell. (F) Tor1 mediates resistance to caffeine. The indicated wild type and mutant strains were grown to log phase and plated on media containing 10 mM caffeine or 5 µM Torin1.

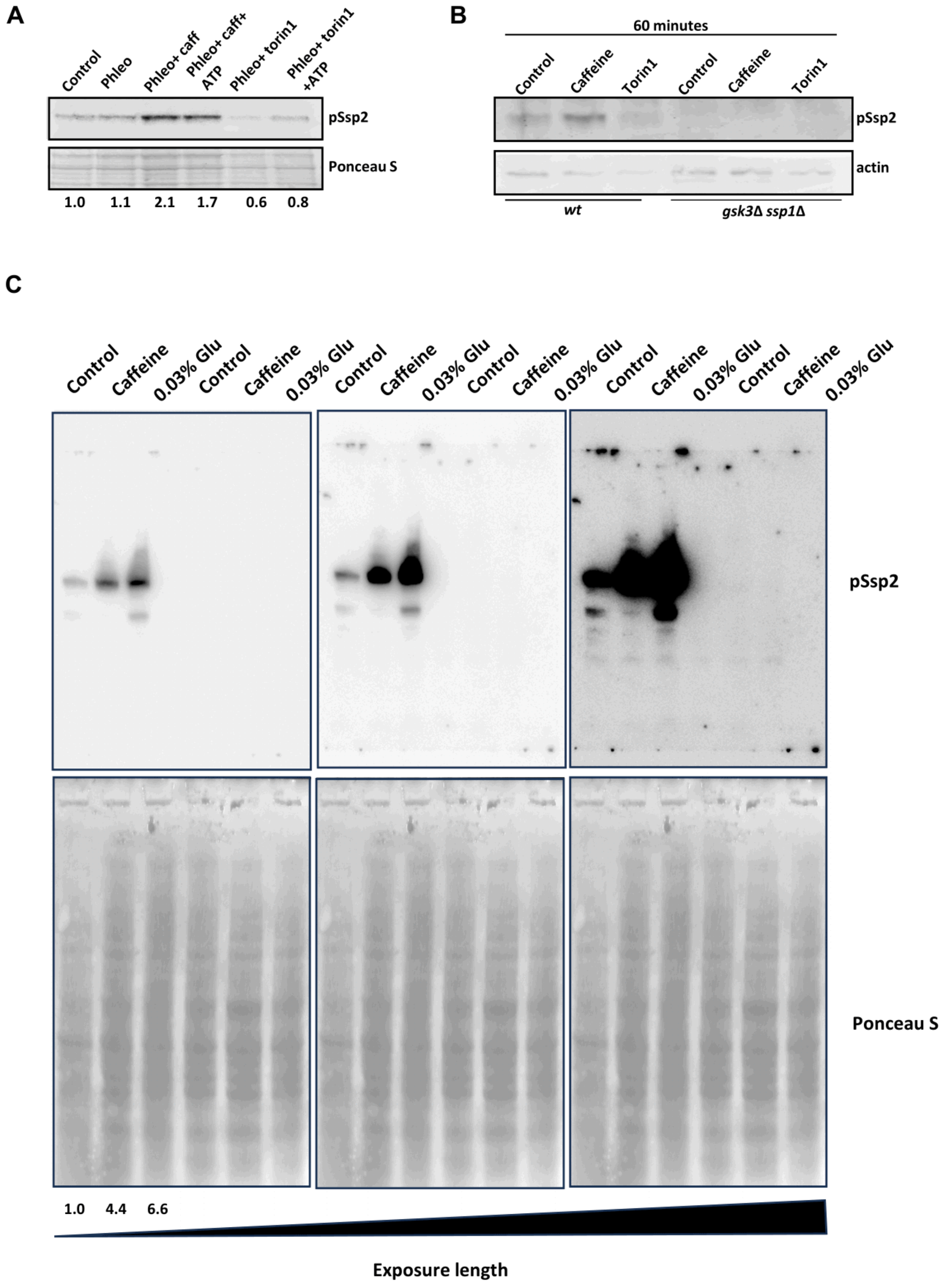

Supplementary Figure S3 (A). Effect of ATP on caffeine-induced Ssp2T189A phosphorylation. Wild type cells were grown to log phase and exposed to 5 µg/ mL phleomycin for 2 h. Cells were then exposed to 10 mM caffeine or 5 µM Torin with or without ATP for another 2 h. Cells were pretreated with 10 mM ATP for 30 minutes. Cell lysates were resolved by SDS-PAGE and membranes stained with antibodies directed against pSsp2 T189A. Gel loading was monitored by ponceau S staining. Quantification was performed using Image J software. (B) Validation of anti- phospho-Ssp2 antibody (Phospho-AMPKα (Thr172) (D4D6D) Rabbit mAb #50081). Wild type and *gsk3*Δ *ssp1*Δ mutants were incubated with 10 mM caffeine or 5 µM Torin1 for 60 minutes as indicated. Total cell lysates were resolved by SDS-PAGE and membranes probed with antibodies directed against phospho-Ssp2 and actin. (C) Wild type (first three lanes in each panel) and ssp2 T189A mutants (last three lanes in each panel) were incubated with 10 mM caffeine or 0.03 % glucose for 2 h. Cell lysates were treated as in A.

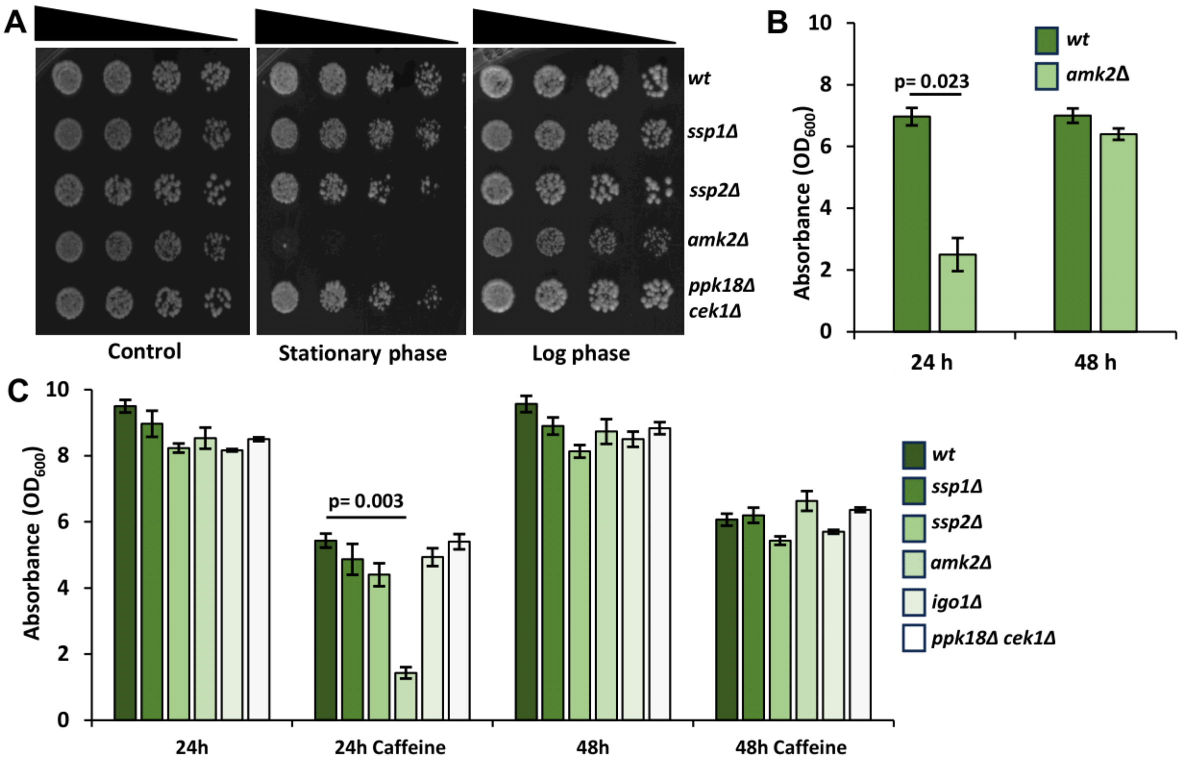

Supplementary Figure 4. Amk2 is required for cell cycle re-entry in the presence of caffeine. (A) The indicated wild type and mutant strains were grown to stationary phase. Cultures were adjusted for cell number, serially diluted 3-fold and plated on YES agar containing 10 mM caffeine. Alternatively, cells were diluted into fresh media and grown to log phase before plating on 10 mM caffeine. (B) Wild type and *amk2*Δ strains were grown to stationary phase and, diluted to an OD_{600} of 0.2 and cultured in YES media containing 10 mM caffeine for 24 h or 48 h. Results represent the mean of at least 3 independent experiments. Error bars represent the mean ±S.E (*n= 3*). (C) Wild type and mutant strains were grown to stationary phase and treated as in B. Results represent the mean of at least 3 independent experiments. Error bars represent the mean ±S.E (*n= 3*).

Supplementary Table S1. List of fission yeast strains used in this study.

Strain	Genotype	Source/Reference
h⁻L972	*h-*	Laboratory stocks
tor1Δ	*h- tor1::kanMX6*	Laboratory stocks/[5]
gad8Δ	*h- gad8:: ura4 ade6- M216 leu1 ura4- D18*	YGRC
ssp1Δ	*h - ssp1::kanMX6*	Moseley laboratory/[34]
ssp1Δ	*h - ssp1::kanMX6*	Laboratory stocks
pom1- GFP ssp1Δ	*pom1-GFP-kanMX6 ssp1Δ::ura4 ura4-D18*	Bahler laboratory
ssp2Δ	*h - ssp2::kanMX6, h + ssp2::kanMX6*	Moseley laboratory and Laboratory stocks/[34]
amk2Δ	*h - amk2::kanMX6*	Moseley laboratory/[34]
gsk3Δ	*h– gsk3::kanMX6*	Laboratory stocks/[26]
gsk3Δ ssp1Δ	*h– ssp1::kanMX6 gsk3::hphMX6*	Laboratory stocks/[26]
gsk3Δ ssp2Δ	*h– ssp2::kanMX6 gsk3::hphMX6*	Laboratory stocks/[26]
gsk3Δ amk2Δ	*h– amk2::natMX6 gsk3::hphMX6*	Laboratory stocks/[26]
ppk18Δ	*ppk18Δ::KanMX6 h+*	Moseley laboratory
cek1Δ ppk18Δ	*h+ ppk18::KanMX6 cek1::ura4+ ura4-D18*	Moreno laboratory
cek1Δ ppk18Δ	*h- Δcek1::natMX Δppk18::hphMX*	Takeda laboratory
igo1Δ	*h- igo1Δ::KanMX6*	Moseley laboratory/[34]
rhp6Δ	*h+ ade6-M210 leu1-32 ura4-D18 rad16::ura4+*	YGRC⋆
cut8Δ	*h+ Δcut8::ura4+*	YGRC⋆
sck1-3HA	*h+ sck1-3HA:natMX6*	YGRC⋆
sck1- 3HA	*h-sck1+* -3HA: hphMX	Nakashima laboratory
sck2- 3HA	*h-sck2+* -3HA: hphMX	Nakashima laboratory
ssp2- Myc	*ssp2*-13myc-hphR *h+*	Moseley laboratory/[34]
ssp2 T189A	h- ssp2::ura4+ ura4-d18 leu1-32::pJK148- ssp2T189A	Moreno laboratory
cut8- 8Myc	*h- leu1 cut8+- myc8*	YGRC
Maf1.pk	*maf1.pk::KanMX6*	Petersen laboratory/[15]
cdc25- GFP	*cdc25-GFPint cdc25:: kanMX6 ura4-D18 leu1-32*	Young laboratory
cdc25 (12A)- GFP	*cdc25(12A)-GFPint cdc25:: kanMX6 ura4-D18 leu1-32*	Young laboratory

⋆Yeast Genetic Resource Center (YGRC) Japan.

REFERENCES

5. Rallis C, Codlin S, and Bähler J (**2013**). TORC1 signaling inhibition by rapamycin and caffeine affect lifespan, global gene expression, and cell proliferation of fission yeast. **Aging Cell** 12(4): 563–573. doi: 10.1111/acel.12080

15. Davie E, Forte GMA, and Petersen J (**2015**). Nitrogen Regulates AMPK to Control TORC1 Signaling. **Curr Biol** 25(4): 445–454. doi: 10.1016/j.cub.2014.12.034

26. Rallis C, Townsend S, and Bähler J (**2017**). Genetic interactions and functional analyses of the fission yeast gsk3 and amk2 single and double mutants defective in TORC1-dependent processes. **Sci Rep** 7(1): 44257. doi: 10.1038/srep44257

34. Schutt KL, and Moseley JB (**2017**). Transient activation of fission yeast AMPK is required for cell proliferation during osmotic stress. **MBoC** 28(13): 1804–1814. doi: 10.1091/mbc.e17-04-0235
